# Bibliometric Trends in Open Surgical and Endovascular Cerebrovascular Research

**DOI:** 10.7759/cureus.25204

**Published:** 2022-05-22

**Authors:** Jason Yuen, Mohamed Sobhi Jabal, Luis E Savastano, David F Kallmes

**Affiliations:** 1 Neurological Surgery, Mayo Clinic, Rochester, USA; 2 Radiology, Mayo Clinic, Rochester, USA

**Keywords:** cerebrovascular treatment, endovascular treatment, citations, bibliometrics, altmetric

## Abstract

The last decade has witnessed a major expansion in endovascular interventions concurrent with a contraction of open neurovascular surgeries. Whether research efforts have also shifted from open to endovascular neurosurgery is an effect that has not been explored extensively. Understanding the bibliometric trend is important for researchers, funding agencies, and publishing journals. The aim of this review is to explore this potential shift.

We compared the bibliometrics of open cerebrovascular and endovascular research articles published in two neurosurgical journals (Journal of Neurosurgery, Neurosurgery) and two neuroradiological journals (Journal of Neurointerventional Surgery, American Journal of Neuroradiology). Data were collected between September 26, 2021, and October 18, 2021. Articles published in 2011, 2013, 2015, 2017, and 2019 from the journals were screened. Neurovascular articles were classified into open surgical, endovascular, or mixed. Bibliometric parameters were collected via SCOPUS and journals’ websites. A total of 8,018 articles were screened, of which 1,551 were included (16.2% open, 62.2% endovascular, 21.5% mixed). Most articles were related to aneurysms (76%). Open-access status correlated with increased citations (p<0.001) and Altmetric (p<0.001), which measures online activity. Comparing 2011 and 2019, the article distribution (open/endovascular/mixed) has changed significantly (χ^2^ test, p=0.002), with open articles dropping from 23.6% (68/288) to 12.9% (44/342) and endovascular articles rising from 56.6% (163/288) to 65.8% (225/342). Using the Kruskal-Wallis test, the citation distribution is different across the three groups in 2019 (p<0.001), favoring endovascular articles, but not in the other years. Our study suggests a trend of diminishing open neurovascular research output and increasing endovascular research output, in terms of both the number of articles and the citations. More time for citation accumulation may be required to verify this trend.

## Introduction and background

With advances in minimally invasive endovascular techniques and technologies, the volume of open cerebrovascular surgeries has become increasingly smaller and subspecialized [1]. Initially, for brain aneurysms, this shift was accelerated by trials such as the ISAT (International Subarachnoid Aneurysm Trial) that demonstrated the safety and value of endovascular treatment [2,3]. Data from the national database and large volume centers also showed a marked increase in endovascular treatments for aneurysms relative to open treatments [4,5]. More recently, with emerging evidence of the efficacy of thrombectomy treatments in ischemic stroke, the field of neurovascular therapy has expanded dramatically over the past decade [6-8]. Whether or not research effort has also shifted from open cerebrovascular surgeries toward endovascular interventions is an effect that has not been studied extensively.

This is an interesting and important question. Not only does answering this question help inform researchers of the ongoing trend of research, one (but not the only) metrics in resource allocation for research is also often based on academic productivity, which is important for researchers and funding agencies. In addition, the bibliometric studies provide researchers with objective evidence to assess the impact of their research relative to other publications in a similar domain. Therefore, understanding the trend in research is important for researchers, gatekeepers of research funding, and publishing journals [9].

To better inform academic clinicians and other researchers, in the current study we compare the citation numbers and Altmetric indices (measurement of online activity such as social media) [10] of open surgical and endovascular cerebrovascular research articles published in four prominent journals, of which two are general neurosurgical journals (Journal of Neurosurgery [JNS] and Neurosurgery) and two are neuroradiological journals (Journal of Neurointerventional Surgery [JNIS] and American Journal of Neuroradiology [AJNR]). These four journals were chosen as they are among the most prominent journals in the field with a high volume of published cerebrovascular studies according to the authors’ experience. In addition, they have similar journal impact factors - JNS (5.115), Neurosurgery (4.654), JNIS (5.836), AJNR (3.825) [11] - which makes it a more homogenous comparison.

## Review

Methods

Data were collected between September 26, 2021, and October 18, 2021. Every article from the four journals published in 2011, 2013, 2015, 2017, and 2019 was screened manually by the authors (J.Y., M.S.J.), mostly using the title and abstract. Full articles were assessed if necessary. Articles with a cerebrovascular theme were included, except those that focused on purely diagnostic methods (no interventional component), trauma (including subdural hematomas), oncology, other head and neck pathologies (e.g., mandibular arteriovenous malformations), microvascular decompression, idiopathic intracranial hypertension, radiotherapy treatment (unless in combination with open and/or endovascular interventions), stroke prevention (e.g., inferior vena cava filter insertion), and peripheral intravenous thrombolysis, and those with no open or endovascular interventions. Editorials, letters, conference abstracts, and guidelines were also excluded. Scoping reviews were also excluded (systematic reviews and meta-analyses are included in the study).

Bibliometric data were collected via the SCOPUS electronic database [12] and websites of individual journals [13-16]. Neurovascular articles were classified into open surgical vascular articles, endovascular articles, or mixed. The following data were also collected: year of publication, whether the article is open access (OA), number of citations, Altmetric score, country of corresponding author’s institute, neurosurgical theme of the article, and type of research. OA status can be subclassified in multiple ways [17], e.g., “green” and “gold,” but in the current study, we adopted a pragmatic classification where articles that can be viewed by the public with no extra cost are classified as OA.

Statistical analysis and graph constructions were performed using SPSS 25.0 for Mac (IBM Corp, Armonk, NY, USA). Year-to-year comparison of distribution of articles (open/endovascular/mixed) was compared using χ2 test. Differences in citation number and Altmetric between groups (open/endovascular/mixed; OA vs non-OA) was compared using the Kruskal-Wallis test due to the non-parametric nature of the data. Correlation of citation count and Altmetric was calculated using the two-tailed Spearman rank correlation coefficient (ρ). Statistical significance was set at p<0.05.

Results

Overall, 1,551 articles were selected out of 8,018. A detailed breakdown is shown in Table 1. The majority of articles are endovascular-related (965/1551; 62.2%). In addition, almost half of the articles are related to aneurysm/subarachnoid hemorrhage (760/1551; 49.0%). The majority of articles in all journals are original clinical articles.

**Table 1:** Distribution of articles across the four studied journals. The “case report” category encompasses case reports and case series with less than five patients, and case series with five or more patients are classified as “clinical original.” AJNR, American Journal of Neuroradiology; AVM, arteriovenous malformation; endo., endovascular; JNIS, Journal of Neurointerventional Surgery; JNS, Journal of Neurosurgery; OA, open access; SAH, subarachnoid hemorrhage; SEM, standard error of mean

	Neurosurgery	JNS	AJNR	JNIS	Total
No. of articles screened	2472	2255	2201	1090	8018
No. of articles included					
Open	99 (27.4%)	147 (35.3%)	1 (0.4%)	5 (1.0%)	252 (16.2%)
Endo.	121 (33.5%)	110 (26.4%)	254 (95.8%)	480 (94.3%)	965 (62.2%)
Mixed	141 (39.1%)	159 (38.2%)	10 (3.8%)	24 (4.7%)	334 (21.5%)
Total	361	416	265	509	1551
Average of citations ± SEM (range)	26.5±1.4 (1-192)	23.2±1.6 (0-320)	34.8±2.4 (0-314)	18.2±0.8 (0-146)	24.3±0.7 (0-320)
Average Altmetric ± SEM (range)	1.7±0.2 (0-42)	4.7±0.9 (0-323)	3.2±0.4 (0-74)	5.8±0.8 (0-299)	4.1±0.4 (0-323)
Percentage of OA	8.9%	3.6%	14.0%	14.1%	10.1%
Distribution of topics					
Aneurysm/SAH	211 (58.4%)	237 (57%)	157 (59.2%)	155 (30.5%)	760 (49.0%)
Fistula/AVM	48 (13.3%)	56 (13.5%)	24 (9.1%)	47 (9.2%)	175 (11.3%)
Cavernoma	18 (5.0%)	20 (4.8%)	0 (0%)	0 (0%)	38 (2.5%)
Stroke	24 (6.6%)	17 (4.1%)	78 (29.4%)	257 (50.5%)	376 (24.2%)
Venous thrombosis	3 (0.8%)	7 (1.7%)	0 (0%)	5 (1.0%)	15 (1.0%)
Carotid artery dissection or stenosis	27 (7.5%)	29 (7.0%)	1 (0.4%)	8 (1.6%)	65 (4.2%)
Vertebral artery dissection or stenosis	4 (1.1%)	3 (0.7%)	1 (0.4%)	2 (0.4%)	10 (0.6%)
Moyamoya	19 (5.3%)	30 (7.2%)	0 (0%)	2 (0.4%)	51 (3.3%)
General open	2 (0.6%)	7 (1.7%)	0 (0%)	0 (0%)	9 (0.6%)
General endo.	5 (1.4%)	8 (1.9%)	4 (1.5%)	32 (6.3%)	49 (3.2%)
General mixed	0 (0%)	2 (0.5%)	0 (0%)	1 (0.2%)	3 (0.2%)
Type of article					
Clinical original	296 (82.0%)	320 (76.9%)	223 (84.2%)	377 (74.1%)	1216 (78.4%)
Case report/technical note	30 (8.3%)	58 (13.9%)	4 (1.5%)	100 (19.6%)	192 (12.4%)
Basic science	25 (6.9%)	23 (5.5%)	21 (7.9%)	18 (3.5%)	87 (5.6%)
Systematic review/meta-analysis	10 (2.8%)	15 (3.6%)	17 (6.4%)	14 (2.8%)	56 (3.6%)
Top three most popular countries (corresponding author)	USA (55.1%); Japan (11.4%); Germany (5.5%)	USA (46.6%); Japan (13.9%); China (7.2%)	USA (27.9%); France (15.5%); Germany (13.2%)	USA (59.3%); Germany (7.9%); China (5.3%)	USA (49.6%); Japan (8.1%); Germany (7.7%)

Breaking down into the subspecialty groups (open surgical/endovascular/mixed), the citation count (across all years) does not differ significantly across the group (p = 0.444), but the Altmetric appears to be lower in the endovascular and mixed groups compared to the open surgical group (p<0.001) (Table 2). However, considering the progression over the years, comparing 2011 and 2019 alone, the article distribution (open/endovascular/mixed) has changed significantly (χ^2^ test, p=0.002), with open articles dropping from 23.6% (68/288) to 12.9% (44/342) and endovascular articles rising from 56.6% (163/288) to 65.8% (225/342). A similar trend also applies when considering solely the two neurosurgical journals. Here, the proportion of open articles dropped from 38% (67/176) to 27% (43/161), and the proportion of endovascular articles was approximately constant, changing from 31% (54/176) to 30% (48/161) (χ^2^ test, p=0.034).

**Table 2:** Bibliometric information across the different subspecialty groups. Comparison of citation number, Altmetric, and number of authors was performed using the Kruskal-Wallis test SEM, standard error of mean

	Open	Endovascular	Mixed	p-Value
Average number of citations ± SEM (range)	23.0±1.7 (0-195)	25.1±1.0 (0-314)	23.1±1.6 (0-320)	0.444
Altmetric ± SEM (range)	4.3±1.3 (0-323)	4.4±0.4 (0-299)	3.1±0.4 (0-52)	<0.001
Average number of authors per article ± SEM (range)	6.4±0.2 (1-15)	7.8±0.1 (1-31)	7.3±0.2 (1-25)	<0.001

**Figure 1:**
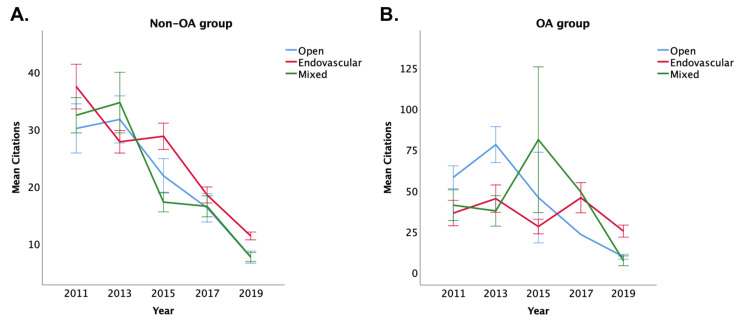
Changes in citation number over time in (A) non-O) group and (B) OA group. OA, open access

**Figure 2:**
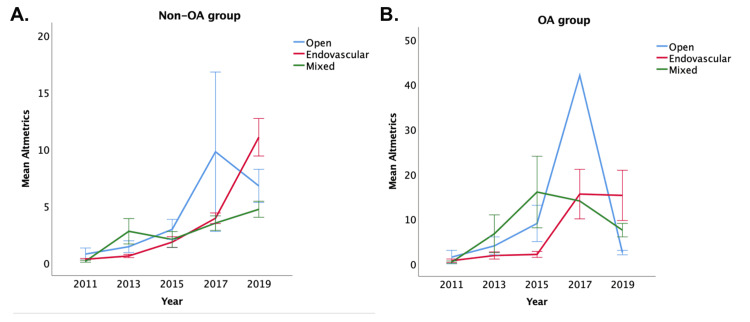
Changes in Altmetric over time in (A) non-OA group and (B) OA group. OA, open access

In addition, with all four journals, using the Kruskal-Wallis test, both the citation distribution and Altmetric score are significantly different across the three groups in 2019 (both p<0.001), favoring endovascular articles, but not in the other years (except the Altmetric score in 2017, which is also statistically significant with p=0.001, favoring endovascular articles). These are demonstrated in Figures 1, 2. The citation count fell over time (as citation takes time to accumulate), but the Altmetric score increased from 2011 to 2019.

**Figure 3:**
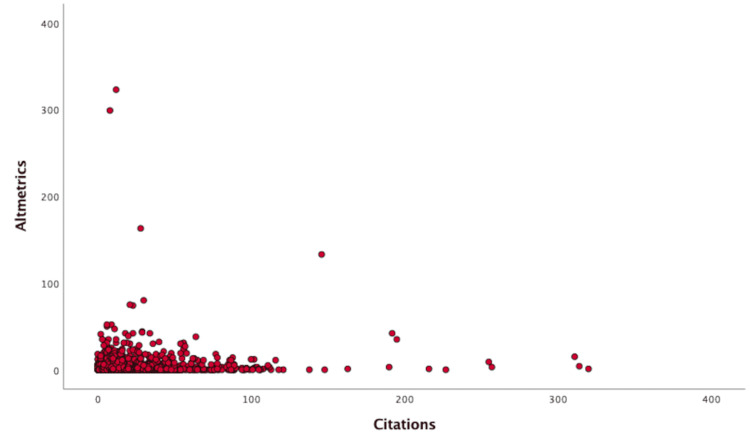
Correlation between citation count per article and Altmetric score.

**Figure 4:**
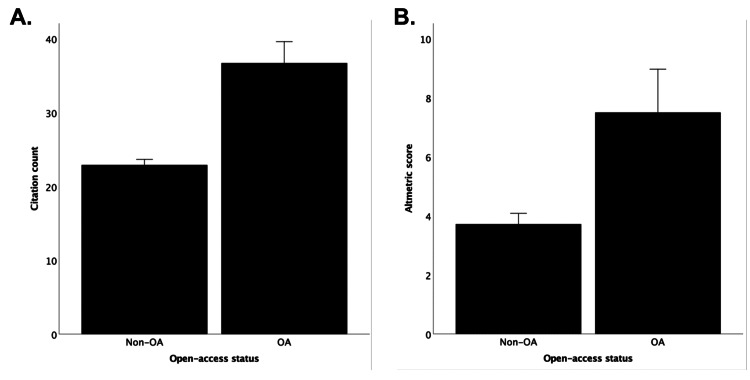
Differences in (A) citation count and (B) Altmetric score between articles of different open-access statuses.

As shown in Figure 3, there is no statistically significant correlation with the Altmetric scores (ρ=-0.025; p=0.326). OA articles have statistically higher citation counts (p<0.001) and Altmetric scores (p<0.001), as demonstrated in Figure 4.

Discussion

Our study adds to the current literature by assessing the relative changes in research focus between open surgical and endovascular cerebrovascular research. Our results suggest a trend where open neurovascular research output is diminishing while endovascular research output is increasing, as evidenced by the changes in publication number. Furthermore, the impact of open surgical research has also reduced relative to endovascular research, as demonstrated by the changes in citation counts and Altmetric scores.

This potential decline in the open surgical research output and impact may be explained by the decline in open vascular case volume and the fact that a large proportion of these cases are now concentrated in a few highly specialized centers [18]. This may lead to challenges such as patient recruitment and generalization of results, and therefore such research is harder to be conducted. For these reasons, a multicenter approach is often adopted, which, however, can be very costly. This is reflected in the fact that most studies are published in more wealthy developed countries and those with high patient volume, such as the USA, Germany, Japan, and China [18].

However, it is difficult to ascertain the temporal relationship on how research may reflect surgical volume, as research output could both be a leading and a lagging indicator of treatment paradigm shift. Early rise in research output may signal an upcoming change in the clinical practice, while once that change has been implemented it may also serve to further increase the research output (for example, increasing number of patients and operators may further popularize the treatment modality).

Another possible explanation for the relative decline in open surgical output and impact may be the relative lack of new open techniques or tools over the last decades (unlike endovascular devices), which couples with the evolving costs of endovascular procedures and explosion of commercial investment in endovascular research. Furthermore, the emergence of dual-trained open/endovascular neurosurgeons and shift in practice from radiology to neurosurgery (especially in the United States) may also be part of the reasons, particularly since there is a large demand for publications among academic neurosurgeons. However, further studies are needed to confirm that.

Altmetric

Citation of an article accumulates over time as it is cited by other publications. Hence, articles published closer to the data collection date tends to have lower citation count. However, interestingly, Altmetric, an algorithm that takes into account activity on the Internet, such as Wikipedia, newspaper, and social media [10], is actually higher in the later years. This is likely to be caused by the increasing popularity of smart phones, as well as social media use such as Twitter [19]. Furthermore, the Altmetric score bears no correlation with the citation count. A previous study suggests that the impact factor, which is based on citation count, is strongly correlated with other newer indices such as SCImago Journal indicator, SCOPUS h-index, Google h-index, Eigenfactor, and Article Influence Score (detailed description of these indices can be found in the study by Yuen [20]). None of these capture the Internet activity in the same way Altmetric does, which provides another dimension to how information is obtained by the audience. Therefore, authors and editors should consider evaluating their research impact using this in conjunction with traditional metrics, especially given its increasing popularity and importance.

Is the cost for open access worth it?

In the current study, we demonstrated that when an article is free to view (sometimes costing the authors a fee or sometimes at the discretion of the editors), their citation counts and Altmetric scores are higher. However, the cost of publishing OA can be very high. With the four journals here, the cost to publish an original article can vary between $750 and $4,269 (although there are discounts available for researchers from developing countries) [13-16]. Given the importance to disseminate research, this should incentivize more governmental support to subsidize researchers in opting for this option. Furthermore, having more citations (and hence a higher h-index) is associated with increased success in the National Health of Institute (NIH) grant application, and therefore there are also reasons for the academic institutes to support OA costs [21].

Limitations

There are certain limitations to this study. In the current study, high profile but more general medical journals such as New England Journal of Medicine and Lancet Neurology were excluded. Although these journals generally have a higher impact factor and important landmark papers are sometimes published there, we do not want them to skew our results. In particular, a small number of recently published endovascular articles (such as the study by Nogueira et al. [22]) in these journals are outliers compared to the majority of the endovascular literature. Inclusion of journals that regularly publish cerebrovascular studies provides a more balanced view. Hence, we decided on the four chosen journals to achieve our objectives since they are all highly regarded neurosurgical/neuroradiological journals with similar impact factors and excellent track record in publication of cerebrovascular studies. Furthermore, the two neuroradiological journals do not publish open cerebrovascular research in general, which is the reason why we assess citation counts and other bibliometric metrics to compare the impact of articles.

The citation counts, particular those in the later years, accumulate over time. Hence, the deduction that endovascular research is overtaking open surgical research probably cannot be confirmed until years later. Nonetheless, the results here are suggestive that this is the case.

We have only sampled the odd number years in this study but given that more than 1,500 articles are included and the general trend is the focus, the risk of significant bias is low on balance. Increasing the number of years in the study would increase the duration of data collection, which can introduce bias as bibliometric data changes over time.

## Conclusions

The current study sampled articles from four prominent journals in the field of neurosurgery and neuroradiology across five years (2011 to 2019). It demonstrated a trend where open neurovascular research output is diminishing while endovascular research output is increasing in terms of the number of articles. Furthermore, considering the citation counts, endovascular research has also been cited progressively more. We have also demonstrated OA status does increase the impact in terms of citations and Altmetric scores, providing incentives for researchers to opt for that option. However, more time for citation accumulation may be required to verify this trend.
